# Does the pain experienced during orthodontic treatment and bracket removal depend on the architecture of the bracket or debonding method?

**DOI:** 10.1093/ejo/cjae073

**Published:** 2024-12-12

**Authors:** Marta Gibas-Stanek, Piotr Fudalej

**Affiliations:** Department of Prosthodontics and Orthodontics, Dental Institute, Faculty of Medicine, Jagiellonian University Medical College, Montelupich St. 4/108, 31-155 Krakow, Poland; Department of Prosthodontics and Orthodontics, Dental Institute, Faculty of Medicine, Jagiellonian University Medical College, Montelupich St. 4/108, 31-155 Krakow, Poland; Department of Orthodontics and Dentofacial Orthopedics, School of Dental Medicine, University of Bern, Freiburgstrasse 7, CH-3010 Bern, Switzerland

**Keywords:** orthodontic brackets, pain measurement, dental debonding

## Abstract

**Background:**

The fear of pain during the various stages of orthodontic treatment with fixed appliances is a common concern of patients. Therefore, the present research aimed to thoroughly investigate the impact of bracket architecture on pain perception during active treatment, debonding, and adhesive removal.

**Materials:**

One hundred consecutive patients who completed treatment with one of two bracket systems (2-slot brackets with an integral base or conventional twin brackets with foil mesh) were included in this prospective cohort study. Participants were asked to evaluate the level of pain encountered throughout their orthodontic treatment with the fixed appliances and during bracket and adhesive removal, utilizing a 0–10 numerical rating scale. Two different methods of bracket removal (bracket debonding pliers and Lift-Off Debonding Instrument) and adhesive removal (adhesive removal pliers and rotary instrument) were tested.

**Results:**

Our study found moderate and comparable levels of pain during active treatment in both groups (4.4 ± 1.6 in the 2-slot group and 3.9 ± 1.9 in the Twin group). Debonding of brackets with integral base caused more discomfort compared to conventional twin brackets and using bracket removal pliers elicited more pain sensations than when Lift-Off Debonding Instrument were employed. Patients are likely to prefer adhesive removal methods involving rotary instruments despite the sound and vibrations produced by contra-angle handpiece.

**Limitations:**

The lack of randomization in patient grouping introduces an increased risk of bias.

**Conclusions:**

The results of the present study suggest that the bracket architecture, particularly the construction of the bracket base, affects the level of discomfort experienced during debonding.

**Trial registration:**

ClinicalTrials.gov, NCT06324162, Registered 20 March 2024—Retrospectively registered, https://clinicaltrials.gov/study/NCT06324162

## Introduction

Pain and discomfort during orthodontic treatment stem from compression and tension within the periodontium [1]. These sensations typically begin shortly after the insertion of orthodontic appliances, rapidly increasing within the initial hours and peaking on the first-day postinsertion. Subsequently, they gradually subside over the course of the first week [2]. Various factors contribute to this experience, with individual susceptibility being a key factor [3, 4]. Additionally, the severity of pain is influenced by variables such as sex, age, emotional state, and the magnitude of applied force [1].

To provide more patient-oriented care, various patient-reported outcome measures (PROMs) were developed and targeted on a specific disease. For dental research and clinical practice, oral health-related quality of life (OHRQoL) has important implications thanks to a comprehensive representation of the individual’s subjective perspective. It comprises an assessment of oral health and function, social and emotional status as well as treatment satisfaction [5].

Recent research, as highlighted in a systematic review, indicates that the type of orthodontic appliance also affects the intensity of pain [6, 7]. A comparison between patients treated with clear aligners and those treated with fixed appliances revealed greater oral health-related quality of life (OHRQoL) for aligner-treated individuals, although this difference was not sustained by the end of treatment [8]. Among fixed appliances, self-ligating and conventional brackets produced comparable levels of pain intensity [9, 10].

While the onset of pain and discomfort is most pronounced at the beginning of treatment with fixed appliances, the removal of brackets can also lead to discomfort [11, 12]. To mitigate the perception of pain during the debonding procedure, various methods and adjuncts can be employed. For instance, applying intrusive biting force, such as biting on a cotton roll, may reduce pain by stabilizing the teeth and counteracting the debonding forces on the periodontal ligament [13]. The choice of debonding instrument is another factor influencing pain perception; research by Pithon *et al*. [14] revealed that patients reported less pain when a lift-off debonding instrument (LODI) was used than when a ligature-cutting device was used.

In theory, the design of orthodontic brackets, encompassing elements such as shape and size, along with the inherent physical properties of the bracket system—such as stiffness, pliability, rigidity, and others—has the potential to impact the experience of pain and discomfort throughout both the treatment and debonding processes [15]. For instance, the design of a double-slot bracket, exemplified by Cannon Ultra® (G&H, Franklin, IN, USA) [16], differs significantly from that of a conventional twin bracket such as a Magnum bracket (G&H, Franklin, IN, USA). The Cannon Ultra® bracket, featuring two slots, combines the properties of a standard edgewise system with the low friction characteristics associated with the Begg technique. While the insertion of the archwire into the low-friction slot may alleviate pain perception in the initial phase of treatment, the additional slot and gingival tie wing on the bracket’s shape may render it potentially less comfortable during treatment than the twin bracket.

In the context of debonding, the force applied to the bracket base and transmitted to the tooth introduces transitory stress to the periodontium. Previous research has indicated that the force required to detach the bracket depends, in part, on the architecture of the bracket base and the direction of the force [17]. The integral base with anchor pylons in the double-slot Cannon Ultra system offers superior adhesive retention compared to traditional foil mesh, necessitating greater forces for debonding one-piece brackets [18, 19]. Additionally, the shear bond strength exerted by traditional bracket removal pliers (BRP) tends to be greater than the tensile force applied when using an LODI plier [20].

The removal of residual adhesive can also contribute to pain and discomfort. Previous studies have demonstrated that LODI produces separation at the bracket-adhesive interface, while BRP tends to break the adhesive closer to the enamel, resulting in varying amounts of adhesive remaining on the enamel, requiring cleaning [21, 22]. Manual techniques, such as the use of adhesive removal pliers (ARPs) without rotary tools, are generally more socially accepted than micromotor or contra-angle handpieces. However, the impact of this technique on the perception of pain and discomfort remains unclear.

This study had three aims: (i) to determine the effect of bracket architecture (double-slot bracket versus conventional twin bracket) on pain perception during active orthodontic treatment (the H_0_ hypothesis assumed no difference between bracket systems); (ii) to determine the effect of bracket architecture (double-slot bracket with integral base versus conventional twin bracket with foil mesh) on pain perception during bracket removal (the H_0_ hypothesis assumed no difference between bracket systems); and (iii) to determine the effect of the adhesive removal method (manual versus rotary instrument) on pain perception (the H_0_ hypothesis assumed no difference between adhesive removal methods).

## Materials and methods

The research protocol of this clinical study was approved by the bioethics committee of Jagiellonian University (number 1072.6120.196.2021, 29 September 2021). All patients enrolled in the study or their legal guardians were given a detailed description of the experiment and signed an informed consent form.

### Study design

This was a prospective cohort study with two groups of consecutively treated patients.

### Sample size

The minimum sample size was calculated using the following assumptions: α = 0.05, power = 95%, effect size (i.e. detected difference in pain and discomfort perception) = 1, and standard deviation of pain and discomfort perception following debonding = 1.1, as reported by Pithon *et al*. [14]. The estimated minimum sample size was 33 per group.

### Subjects

Fifty consecutive patients of the University Dental Clinic who completed comprehensive orthodontic treatment with a fixed orthodontic appliance with double slot brackets (2-slot group) and 50 consecutive patients who completed comprehensive orthodontic treatment with twin brackets (Twin group) were included in the study based on the following inclusion criteria:

- older than 12 years at the initiation of treatment
- fixed appliance in both dental arches
- Brackets were bonded using the Transbond XT adhesive system (3M Unitek®, St. Paul, USA) following the manufacturer’s instructions.

The exclusion criteria were as follows:

- periodontal disease
- restorations or caries on the buccal surfaces of the teeth
- previous treatment with fixed appliance
- consumption of analgesics within eight hours preceding the debonding appointment.

### Bracket system description

**Figure 1.**
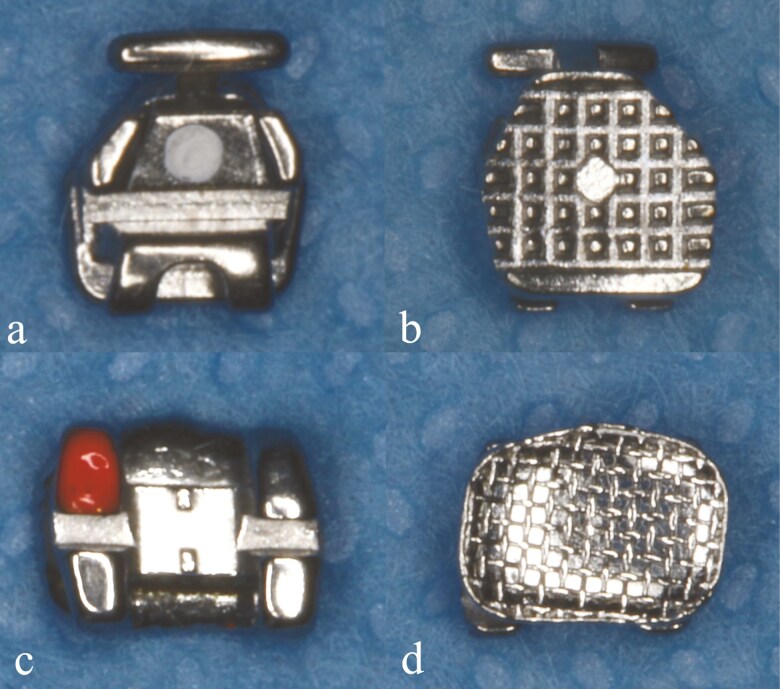
The architecture of brackets tested in the study: 1a, 1b - 2-slot bracket with anchor pylon base (Cannon System), 1c, 1d- traditional twin bracket with foil mesh (Magnum).

The brackets differ significantly in terms of their body and base design. Twin brackets are composed of two pairs of tie wings and one slot, while double slot brackets consist of three tie wings (two occlusal and one gingival) along with two archwire slots—a low-friction ‘wing slot’ and a traditional ‘edgewise slot’ (Fig. 1). The pronounced gingival tie wing in double-slot brackets may lead to irritation of the cheeks and lips, potentially compromising the comfort of orthodontic treatment compared to therapy with twin bracket systems. Conversely, the use of a low-friction slot in double-slot brackets could contribute to lower force levels, alleviating pain sensations associated with periodontal stress during treatment.

Moreover, the bracket systems diverge in the design of their bases. Twin brackets employ a foil mesh to enhance adhesive retention, while the base of double slot brackets is augmented by anchor pylons, creating irregular areas for the adhesive. Additionally, the base of double slot brackets is characterized by increased thickness and rigidity compared to those of twin brackets. These variations may have implications for bond strength, the force required for bracket detachment, and the residual adhesive left on the enamel.

The standardized treatment protocol has been employed for both groups of bracket systems: leveling and niwelization with round NiTi archwires followed by rectangular archwires, space closure using sliding mechanics on rectangular stainless steel archwires, finishing with rectangular or round stainless steel or rectangular TMA archwires. In all the cases intermaxillary elastics were administered to solve sagittal or transverse problems, or reinforce anchorage.

### Bracket removal methods

**Figure 2.**
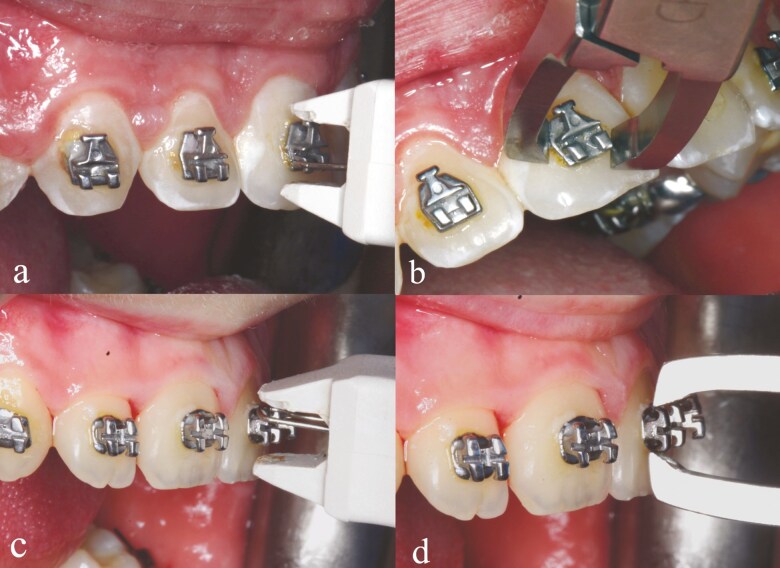
Debonding procedure for the Cannon Ultra 2-slot (a, b) and Magnum twin brackets (c, d).

Brackets on the right side of both dental arches were debonded using the LODI plier (3M Unitek®, St. Paul, MN, USA), while on the left side, the BRP plier (Chifa B. Braun Melsungen AG, Melsungen, Germany) was employed. Initially, the archwires, ligatures, and auxiliaries were removed. As illustrated in Fig. 2, the metal hanger of the LODI was positioned under the gingival tie wing of the twin brackets and the occlusal tie wing in the case of double slot brackets. The instrument was then allowed to rest on the tooth, and compression of the handles facilitated bracket detachment when a pulling force was applied. In the case of debonding twin brackets with the BRP plier, the plier was carefully positioned occlusogingivally between the bracket base and the tooth surface, and gentle squeezing was applied. For the double-slot brackets, the pliers were placed mesio-gingivally and disto-incisally in an oblique orientation.

### Adhesive removal methods

The residual adhesive was removed through two methods: ARPs (Chifa B. Braun Melsungen AG, Melsungen, Germany) were employed in the upper arch, while a carbide tungsten bur (Kerr Corporation, Brea, CA, USA), featuring a working surface length of 4 mm, was utilized with a 1:5 contra-angle handpiece (KaVo EXPERTmatic E25 L, Charlotte, NC, USA) operating at a speed of 12,000, and air cooling was applied in the lower arch.

### Pain and discomfort assessment

Prior to the removal of brackets, participants were administered a brief 2-question survey. In the first question, patients were asked to evaluate the level of pain and discomfort encountered throughout their orthodontic treatment with fixed appliances utilizing a 0-10 horizontal numerical rating scale (NRS), where ‘0’ denoted ‘no pain’, and ‘10’ represented ‘the worst pain imaginable’. The second survey item prompted participants to identify the primary reason for pain during active treatment from the following options: (i) irritation of soft tissues (cheeks, lips, tongue) caused by elements of the fixed appliance, (ii) pain of teeth after insertion or activation of the appliance, (iii) discomfort related to wearing intermaxillary elastics, or (iv) another reason. The questionnaire was on paper and the researcher provided assistance to patients in completing the questionnaire, addressing any inconsistencies or uncertainties.

Throughout the debonding procedure, patients were asked to rate the pain experienced using the NRS after the removal of each bracket. Additionally, pain or discomfort during adhesive removal was evaluated immediately after cleaning in each quadrant, employing the same 10-point NRS scale.

**Figure 3.**
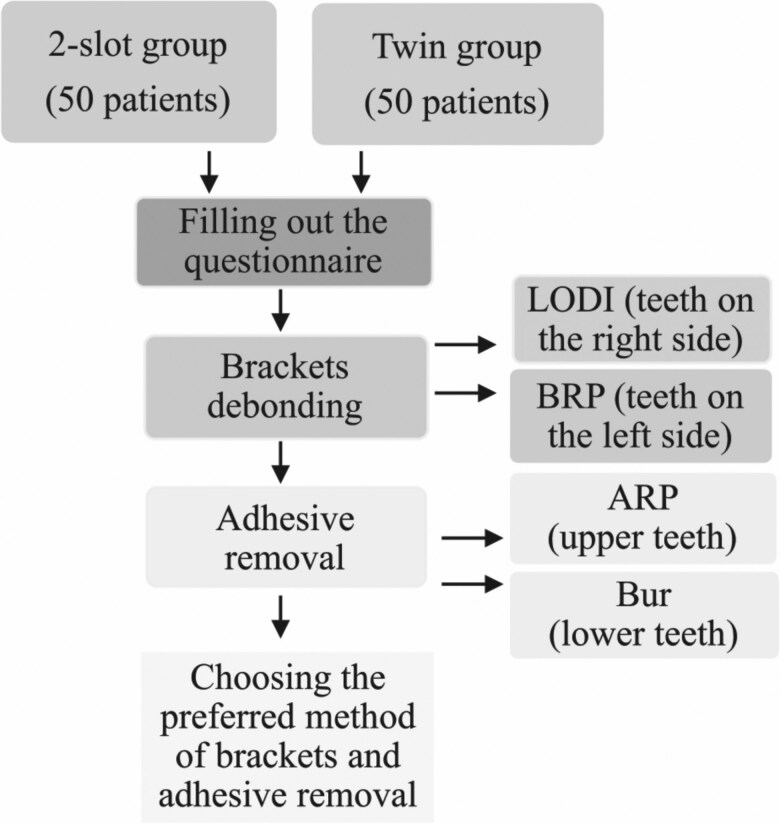
The flowchart of the study.

Ultimately, patients were queried about their preference: which method of bracket debonding and adhesive removal they would choose if the appliance were to be removed a second time? The flowchart of the study is presented in Fig. 3.

All debonding procedures were performed by the same investigator with 8 years of experience in orthodontics (MGS).

### Statistical analysis

Analysis of quantitative variables was performed by calculating the mean, standard deviation, median, and quartiles. Qualitative variables were analyzed by calculating the number and percentage of occurrences of each value. The Mann‒Whitney test was used to compare quantitative variables between two groups. Comparisons of qualitative variables in groups were conducted with the chi-squared test or Fisher’s exact test (when low expected values occurred). The relationships between quantitative variables were assessed with multiple linear regression analysis. The significance level for all the statistical tests was set to 0.05.

Data analysis was performed using R software version 4.3.1 (R Core Team 2023: A language and environment for statistical computing. R Foundation for Statistical Computing, Vienna, Austria).

## Results

### Demographics

A cohort of 100 consecutive patients who underwent treatment with either double-slot brackets (2-slot group) or traditional twin brackets (Twin group) is described in Table 1. No significant differences were observed in the distribution of sex or the mean age of patients between the two groups.

**Table 1.** Descriptive statistics of the participants.

Parameter		2-slot group (*N* = 50)	Twin group (*N* = 50)	*P* value
Sex	Female	29 (58%)	31 (62%)	.838
	Male	21 (42%)	19 (38%)	
Age	Mean (SD)	16.9 (5.5)	16.2 (3.5)	.517
	Median (quartiles)	15.6 (14.9–17.5)	15.4 (14.4–16)	
	Range	13–50	12.3–32.8	
	N	50	50	

*P*—Qualitative variables: chi-squared or Fisher’s exact test. Quantitative variables: Mann-Whitney test.

### Pain and discomfort during orthodontic treatment

The mean pain scores experienced during the active phase of treatment were comparable between the two groups (Table 2).

**Table 2.** Pain levels experienced during the active phase of the treatment.

Group	*N*	Pain during orthodontic treatment							*P* value
		Mean	SD	Median	Min	Max	Q1	Q3	
2-slot	50	4.42	1.60	5	1	7	3	6	.128
Twin	50	3.88	1.88	4	0	8	3	5	

*P*—Mann–Whitney test, SD—standard deviation, Q1—lower quartile, Q3 - upper quartile, * statistically significant (*P* < .05).

In the 2-slot group, the primary source of pain was identified as irritation of soft tissues, whereas patients treated with twin brackets reported the most significant pain occurring after the insertion or activation of the appliance. Statistical analysis using Fisher’s exact test revealed a statistically significant difference between the two groups (Table 3).

**Table 3.** The main cause of pain during active orthodontic treatment.

Cause of pain	Group		*P* value
	2-slot (*N* = 50)	Twin (*N* = 50)	
a) irritation of soft tissues (cheeks, lips, tongue) caused by the elements of the fixed appliance	28 (56%)	13 (26%)	.008
b) pain of teeth after insertion or after activation of the appliance	18 (36%)	32 (64%)	
c) discomfort connected with wearing intermaxillary elastics	4 (8%)	5 (10%)	
d) another reason	0 (0%)	0 (0%)	

*P*—Fisher’s exact test.

### Pain and discomfort during debonding

**Figure 4.**
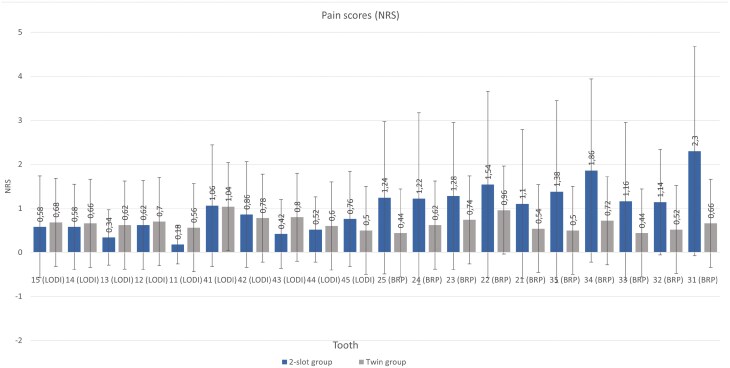
Graphical presentation of mean pain levels experienced during bracket removal with LODI and BRP in the 2-slot group and Twin group.

Figure 4 and Table 4 demonstrate the mean pain levels experienced during debonding in the 2-slot and Twin groups with LODI and BRP pliers. Significantly greater pain scores were recorded during bracket removal with BRP in the 2-slot group (*P* < .001). Debonding with the LODI resulted in similar pain levels in both groups (*P* = .196).

**Table 4.** Mean NRS scores recorded during brackets removal according to the method used.

Group	Debonding pliers	Pain during bracket removal							*P*
		Mean	SD	Median	Min	Max	Q1	Q3	
2-slot	LODI	0.59	1.00	0	0	6	0	1	.196
Twin		0.69	1.07	0	0	5	0	1	
2-slot	BRP	1.42	1.91	1	0	10	0	2	<.001*
Twin		0.61	1.07	0	0	7	0	1	

*P*—Mann–Whitney test, SD—standard deviation, Q1—lower quartile, Q3—upper quartile, * statistically significant (*P* < .05).

### Pain and discomfort during adhesive removal

The use of rotary instruments for adhesive removal was associated with lower NRS scores than the use of ARPs. Additionally, patients in the 2-slot group reported significantly greater pain levels during adhesive cleaning than did those in the Twin group (Table 5).

**Table 5.** Mean NRS scores recorded during adhesive removal according to the method used.

Group	Removal method	Pain during adhesive removal							
		Mean	SD	Median	Min	Max	Q1	Q3	*P*-value
2-slot	Tungsten bur	1.67	1.73	1	0	8	0	2	.008*
Twin		1.08	1.35	1	0	7	0	2	
2-slot	ARP	2.9	2	3	0	9	1	4	.019*
Twin		2.24	1.73	2	0	7	1	4	

*P*—Mann-Whitney test, SD—standard deviation, Q1—lower quartile, Q3—upper quartile, * statistically significant (*P* < .05).

**Figure 5.**
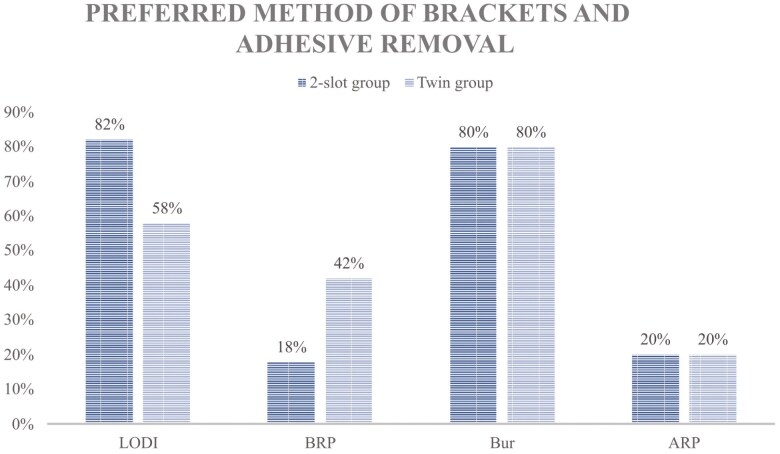
Preferred method of bracket and adhesive removal.

As depicted in Fig. 5, the majority of patients in both groups expressed a preference for bracket debonding with LODI and mechanical adhesive removal as the preferred methods if the brackets were to be removed again.

### Associations between pain levels and sex and age

Multiple linear regression analysis (Table 6) revealed a significant decrease of 0.022 points in pain during debonding for every year of life. While males generally exhibited lower pain levels, a statistically significant difference was observed only for brackets and adhesive removal.

**Table 6.** Multiple linear regression analysis to assess the association between pain levels and sex and age.

Pain during active treatment					
Trait		Parameter	95%CI		*P*
Age		0.016	−0.06	0.092	.683
Sex	Female	ref.			
	Male	−0.06	−0.767	0.646	.868
Group	2-slot	ref.			
	Twin	−0.532	−1.226	0.162	.136
Pain on brackets removal					
Trait		Parameter	95%CI		P
Age		−0.022	−0.035	−0.009	.001*
Sex	Female	ref.			
	Male	−0.217	−0.337	−0.097	<.001*
Group	2-slot	ref.			
	Twin	−0.376	−0.494	−0.258	<.001*
Method	BRP	ref.			
	LODI	−0.375	−0.491	−0.259	<.001*
Pain on adhesive removal					
Trait		Parameter	95%CI		P
Age		−0.014	−0.053	0.024	.462
Sex	Female	ref.			
	Male	−0.685	−1.043	−0.327	<.001*
Group	2-slot	ref.			
	Twin	−0.665	−1.016	−0.313	<.001*
Method	ARP	ref.			
	Tungsten bur	−1.198	−1.528	−0.867	<.001*

*P*—multiple linear regression.

## Discussion

A common concern of patients undergoing orthodontic treatment is the fear of pain during the various stages of treatment with fixed appliances. This fear can be so pronounced that patients with pressing orthodontic needs may choose to delay or refuse treatment altogether. Therefore, it is crucial to thoroughly investigate various aspects related to patient perceptions of pain and discomfort at all stages of treatment. Given the lack of sufficient data in the literature, our research aimed to investigate the impact of bracket architecture on pain perception during active treatment, debonding, and adhesive removal.

Two bracket types were tested in this study: classic twin brackets composed of two pairs of tie wings and one slot and double slot brackets consisting of three tie wings (two occlusal and one gingival) along with two archwire slots—a low-friction ‘wing slot’ and a traditional ‘edgewise slot’. Twin brackets employ a foil mesh at their base, while the base of double slot brackets is augmented by anchor pylons to enhance adhesive retention.

Our results indicate that bracket architecture is associated with pain and discomfort experienced during bracket removal. Specifically, debonding of brackets with anchor pylons (2-slot group) caused more discomfort than debonding of conventional twin brackets with foil mesh, leading us to reject our H_0_ hypothesis. This can be attributed to the greater shear force required to detach the bracket with an anchor pylon base in contrast to a traditional bracket with a retention foil mesh, resulting in increased pain and discomfort [19, 23, 24]. In other words, the bracket base stiffness and resistance to deformation require more force to be removed, which leads to increased pain and discomfort. This explanation likely extends to the observation that using BRP pliers for debonding elicited more pain sensations than did using LODI pliers. *In vitro* experiments support this, suggesting that the minimum tensile force exerted by the LODI necessary to detach the bracket is smaller than the shear force produced by the BRP [25, 26]. Patients also reported less discomfort when debonding with the LODI than when debonding with ligature-cutting pliers [14, 27]. However, it is worth noting that the median pain and discomfort experienced with both tested bracket systems were close to the ‘no pain’ end of the NRS. Therefore, while statistically significant differences were detected, their clinical significance may be negligible.

After the debonding of brackets, the removal of composite material is typically performed to restore the natural smoothness of the enamel surface. While manual methods, which do not involve rotary instruments, are generally preferred in the management of caries removal [28, 29], our findings indicated that adhesive removal with ARP was associated with significantly greater pain levels in both groups. Thus, our other H_0_ hypothesis was rejected. A limited number of reports evaluating orthodontic composite removal with ARP have focused primarily on side effects to the teeth, such as increased enamel roughness or persistent composite remnants [30–32]. Despite the unpleasant sound effects and vibrations associated with the use of a contra-angle handpiece, 80% of participants expressed a preference for adhesive removal with a bur if brackets were to be removed again. Furthermore, higher pain scores were recorded in the 2-slot group, possibly due to the thicker layer of composite remaining on the enamel. Although Atashi *et al*. suggested that anchor pylons provide superior retentive properties for adhesive within the bracket base compared to mesh-base brackets and that bond failure often occurs between the adhesive and enamel [18], the authors did not consider the thickness of the remaining composite material. Clinically, it is often observed that the layer of composite remaining after removing brackets with foil mesh is noticeably thinner than that in the case of anchor pylons, which could explain the greater pain scores in the 2-slot group.

Numerous studies have investigated orthodontic pain during the initial phase of treatment [33–38]; however, little is known about the overall discomfort experienced throughout the treatment process. Our study revealed moderate and comparable levels of pain and discomfort in both groups (4.4 ± 1.6 in the 2-slot group and 3.9 ± 1.9 in the Twin group), which aligns with the findings of Poudel *et al*. (mean pain score 5.1 ± 2.1), who assessed pain perception during the first month of orthodontic treatment with metal brackets [34]. While our study revealed similar overall pain levels during active treatment in both groups (H0 failed to be rejected), participants reported less common soreness of teeth caused by placement or activation of the appliance with 2-slot brackets. Previous studies comparing appliances with traditional wire ligation to a passive self-ligating system (Damon 2™) have shown reduced pain in the Damon group [39–41], which was attributed to incomplete archwire engagement within the passive self-ligating bracket. The insertion of the archwire into the low-friction slot of the 2-slot bracket during the initial phase of leveling might reduce forces acting on teeth due to increased distances between brackets. Additionally, engagement of the archwire in the wing slot decreases undesired moments and forces typically acting on neighboring teeth compared to ligation in the edgewise slot. However, 2-slot brackets were more likely to irritate soft tissues, which could be related to their specific architecture.

In contrast to sex- and age-dependent perceptions of pain and discomfort during debonding and adhesive removal, the level of pain experienced during active treatment was not influenced by the age or sex of the patients. This observation aligns with findings from Lin *et al*., who assessed orthodontic pain over a 3-day period following adjustment appointments [37]. Conversely, Scheurer *et al*. reported that girls perceived general pain intensity to be significantly greater [36]. Our findings showing that male patients experienced lower levels of pain during debonding and adhesive removal are in line with the report of Kilinç *et al*., who also noted lower pain scores among males during debonding, although the difference was not statistically significant [42]. We did not find any studies analyzing the relationship between age and pain resulting from bracket debonding or adhesive removal. Our findings, however, suggest that pain perception reported during debonding decreases with the age of the patient.

Considering practical implications of the present study, it is not recommended to use 2-slot brackets in patients prone to soft tissue irritation (e.g. recurring aphthous ulcers). In the case of debonding brackets with anchor pylon base, since bracket removal pliers elicited two times more pain than LODI, the latter should be treated as the method of choice. This might be of a particular importance when treating patients with a low pain threshold. The manual method of adhesive removal was viewed negatively by most of the study participants. From the operator’s perspective, composite removal with the pliers is more time-consuming and less precise, when compared to the rotary method. In the face of the above observations, adhesive removal pliers should be only considered in patients with teeth sensitivity, where air cooling of rotary handpiece triggers acute dentin pain reaction. Finally, since pain values obtained during brackets and adhesive removal were significantly lower than the pain experienced on the course of active treatment, we are empowered to reassure patients who are afraid of discomfort during the disassembly of the fixed appliance.

While this research provides insights into the debonding process of traditional twin mesh-base brackets and 2-slot brackets with anchor pylons, it is important to acknowledge certain limitations. First, the lack of randomization in patient grouping introduces an increased risk of bias. It is possible that 2-slot brackets were more frequently selected for patients with more severe crowding, potentially leading to a greater perception of pain during active treatment. Second, we lacked information on bracket failure and reattachment during active treatment. In some instances, brackets were sandblasted and reused, which may affect bond strength [43] and consequently the magnitude of pain experienced during debonding. Also, although the bracket bonding and debonding procedures were standardized, differences such as the adhesive thickness may have varied, potentially introducing bias. Third, we did not account for individual pain threshold and pain-modulating factors such as low motivation for orthodontic treatment or elevated dental anxiety levels [3]. Last, the evaluation of pain and discomfort perception during active treatment was conducted at the debonding appointment, which may have led to patients underestimating their actual pain and discomfort levels.

The preliminary findings from this research should be validated by future studies in this direction. Patient randomization and longitudinal observation of the discomfort during active treatment with fixed appliance could provide more accurate data on the subjective perception of pain. Other pain-modulating factors, such as individual pain threshold, psychological status, and motivation for treatment, need to be taken into consideration when designing future research in this field.

## Conclusions

Considering the limitations of this study, the following conclusions are drawn:

1. The bracket architecture, particularly the construction of the bracket base, affects the level of discomfort experienced during debonding. It is not recommended to use 2-slot brackets in patients prone to soft tissue irritation.
2. Patients are likely to prefer adhesive removal methods involving rotary instruments.
3. Bracket debonding and adhesive removal were associated with some pain, but the magnitude of pain was significantly lower than that experienced during the active phase of treatment. Therefore, we can assure the patient that the discomfort associated with the debonding will not be significant.

## Data Availability

The data that support the findings of this study are available from the corresponding author (MGS) upon reasonable request.
