# Correction to: Does the pain experienced during orthodontic treatment and bracket removal depend on the architecture of the bracket or debonding method?

**DOI:** 10.1093/ejo/cjaf016

**Published:** 2025-03-18

This is a correction to: Marta Gibas-Stanek, Piotr Fudalej, Does the pain experienced during orthodontic treatment and bracket removal depend on the architecture of the bracket or debonding method? *European Journal of Orthodontics*, Volume 47, Issue 1, January 2025, https://doi.org/10.1093/ejo/cjae073

In the version originally published, the following affiliation was omitted from the second author’s listing, and has been added: “

Department of Orthodontics and Dentofacial Orthopedics, School of Dental Medicine, University of Bern, Freiburgstrasse 7, CH-3010 Bern, Switzerland.”

